# Impact of Combined Antiretroviral Treatment (cART) on Latent Cytomegalovirus Infection

**DOI:** 10.3390/v17010076

**Published:** 2025-01-09

**Authors:** Aura Temereanca, Luminita Ene, Gratiela Tardei, Camelia Grancea, Cristian L. Achim, Simona Ruta

**Affiliations:** 1 Virology Department, Carol Davila University of Medicine and Pharmacy, 050474 Bucharest, Romania; aura.temereanca@umfcd.ro; 2 Viral Emerging Diseases Department, Ştefan S. Nicolau Institute of Virology, 030304 Bucharest, Romania; cgrancea@yahoo.co.uk; 3 HIV/AIDS Department, Victor Babes Clinical Hospital for Infectious and Tropical Diseases, 030303 Bucharest, Romania; lumiene@yahoo.com (L.E.); gtardei@spitalulbabes.ro (G.T.); 4 Pathology Department, University of California at San Diego, La Jolla, CA 92093, USA; cachim@health.ucsd.edu

**Keywords:** HIV, CMV, antiretroviral therapy

## Abstract

Cytomegalovirus infections and reactivations are more frequent in people living with HIV (PLWH) and have been associated with increased risk of HIV progression and immunosenescence. We explored the impact of combination antiretroviral therapy (cART) on latent CMV infection in 225 young adults parenterally infected with HIV during childhood. Anti-CMV IgG antibodies were present in 93.7% of participants, with lower levels correlating with longer cART exposure and better immunologic parameters. Patients with immunological treatment success (CD4 > 350 cells/mL) had significantly lower CMV IgG titers compared to those with suboptimal immune response to cART. In total, 78% of the tested patients had robust CMV-specific T-cell responses, measured by an IFN-γ release assay. A good immune response to treatment was significantly associated with CMV-specific cellular immunity: IFN-γ level was positively correlated with CD4 and CD8-T cell counts. No differences were observed between patients with suppressed/non-suppressed HIV viremia in terms of CMV humoral and cellular immune response. CMV DNA was detected in only 17% of participants, with lower levels among those with cART-induced immune recovery. The successful antiretroviral treatment with subsequent immunologic reconstitution may lead to restoration of CMV-specific immune responses and effective control of latent infection, limiting episodes of CMV reactivation in HIV-positive individuals.

## 1. Introduction

Following its primary infection, cytomegalovirus (CMV) establishes a lifelong latent infection that usually remains clinically asymptomatic in immunocompetent individuals [1]. Viral latency is maintained in infected hematopoietic stem cells (HSCs) and myeloid progenitors, through cell-specific transcriptional silencing mechanisms. The balance between latency and reactivation is maintained by a complex mechanism involving repression of the viral major immediate–early (MIE) locus, through cell-specific transcription factors and histone regulation [2]. CMV reactivation can occur intermittently, with recurrent viral replication and production of infectious virions, with or without associated clinical symptoms. Immunosuppressed individuals—cancer patients, transplant recipients (solid organ, bone marrow, and hematopoietic cells), and AIDS patients—are at particular risk of severe symptomatic reactivations [3]. People living with HIV (PLWH) are more commonly infected with CMV than the general population [1] and episodes of CMV reactivation are more frequent, mainly due to immune dysfunction, chronic immune activation, and early immunosenescence [3]. Immunosenescence is characterized by an altered T-cell repertoire, with disproportionate accumulation of memory T cells and an exhausted T-cell phenotype, caused by thymus involution and changes in hematopoiesis, that results in impaired immune responses and increased risk of latent virus reactivations [3,4].

In addition, the persistent low-grade inflammation status associated with immune senescence drives an aberrant activation of myeloid cells that harbor latent CMV, with release of infected monocytes into the bloodstream and viral reactivation following their stimulation and differentiation into macrophages or dendritic cells [5]. Conversely, cytomegalovirus infection itself has been associated with inflammation and immune activation [6], increasing the risk for cardiovascular complications [7,8], immunosenescence and aging [9,10], neurocognitive impairments [11], and HIV disease progression [12]. It has been suggested that the immune responses to CMV, rather than the viral replication itself, are responsible for these adverse clinical outcomes [13]. For example, higher anti-cytomegalovirus immunoglobulin G levels have been associated with biomarkers of endothelial injury and carotid intimal media thickness (cIMT) in HIV-positive patients [8] and with worse neurocognitive performance in PLWH [11]. Elevated CMV humoral immune responses were also found to be associated with physical function impairment in HIV-infected middle-aged adults [14].

While combination antiretroviral therapy (cART) including three or more drugs from five different classes of antiretrovirals (nucleoside reverse-transcriptase inhibitors (NRTIs), protease inhibitors (PIs), non-nucleoside reverse-transcriptase inhibitors (NNRTIs), integrase strand transfer inhibitors (INSTIs), and entry inhibitors (Eis)) [15,16] has dramatically decreased the incidence of CMV-related diseases [17], several studies link CMV infection with a suboptimal immune recovery after treatment in HIV-infected patients [18].

We aimed to explore the impact of cART on latent cytomegalovirus infection in a group of chronically HIV-infected young adults from Romania who were infected in the early 1990s in their first years of life via parenteral exposure. We evaluated the association between cART and (1) humoral immune response to CMV measured by circulating anti-CMV IgG antibodies, (2) cellular immune response to CMV measured by an interferon release assay, and (3) asymptomatic CMV replication in peripheral blood mononuclear cells (PBMC).

## 2. Materials and Methods

### 2.1. Patients

We performed a cross-sectional study involving 225 treatment-experienced patients, with life-long HIV infection since early childhood. This study was approved by the ethics committees of the participating institutions, and written informed consent was provided by all participants.

### 2.2. HIV Infection

HIV-1 viral load was assessed with COBAS TaqMan HIV-1 Test Version 2.0 (Roche Molecular System, Inc., Branchburg, NJ, USA), with a lower detection limit of 20 copies of HIV RNA/mL and a linear range between 34 and 10,000,000 copies HIV RNA/mL.

T-cell subsets in the peripheral blood were measured by flow cytometry using the TRIT-EST three-color reagent CD4/CD8/CD3 with TRU-COUNT tubes (Becton Dickinson, San Jose, CA, USA).

### 2.3. CMV Infection

CMV IgM was measured using the CMV IgM capture immunoassay (Dia.Pro Diagnostic Bioprobes, Milano, Italy). A result was interpreted as positive if the sample-to-cut off (S/Co) ratio > 1.2. CMV-specific IgG levels, a marker of chronic latent infection, were measured using a quantitative enzyme-linked immunosorbent assay (Dia.Pro Diagnostic Bioprobes, Milano, Italy). Samples with concentrations > 0.5 IU/mL were considered positive. CMV-specific cellular immunity was tested using a modified QuantiFERON-CMV^®^ (QFT) assay (Cellestis Ltd., Melbourne, Australia), measuring the amount of Interferon-gamma (IFN-γ) released by CD8+ T cells, upon in vitro stimulation with CMV antigens against the test NIL control (absence of peptide stimulation). The Nil value is used to determine if the patient has a preexisting immune response which could cause false positive results. The assay also includes a positive control (mitogen control) where whole blood is stimulated with phytohaemagglutinin. The CMV-cellular immune response was considered: (a) ‘non-reactive’ if (CMV minus NIL control) < 0.2 IU/mL and (mitogen control minus NIL control) ≥ 0.5 IU/mL, (b) ‘reactive’ if (CMV minus NIL control) ≥ 0.2 IU/mL and any value of mitogen control minus NIL control, and (c) ‘indeterminate’ if (CMV minus NIL control) < 0.2 IU/mL and (mitogen control minus NIL control) < 0.5 IU/mL. According to the manufacturer, indeterminate results are not interpretable.

The levels of CMV DNA from peripheral blood mononuclear cells (PBMCs) were measured with the highly sensitive droplet digital PCR assay (ddPCR, Bio-Rad, Hercules, CA, USA), as previously described [19]. DNA was extracted from PBMCs using the QIAamp DNA Mini Kit (Qiagen, Redwood City, CA, USA), and CMV DNA quantification was performed using the following specific primers: forward primer AGGTCTTCAAGGAACTCAGCAAGA, reverse primer CGGCAATCGGTTTGTTGTAAA.

### 2.4. Statistical Analyses

Statistical analyses were performed using SPSS software (v21.0; SPSS Inc., Chicago, IL, USA). The following variables were dichotomized: QFT-CMV (reactive/non-reactive) and HIV RNA (lower/higher than 34 copies HIV RNA/mL; undetectable/detectable). Comparisons were performed using the chi-squared test of independence or Fisher exact text (for sparse data), *t* test (for continuous, normally distributed variables), or Mann–Whitney U test (for continuous, non-normally distributed variables). For all analyses, a *p* value below 0.05 was considered statistically significant.

## 3. Results

### 3.1. HIV+ Group Characteristics

The demographic, virological, and immunological characteristics of the patients are summarized in Table 1. The participants had a median duration of HIV infection of 23.7 years and a median duration of antiretroviral treatment of 12 years. The median time of exposure to the current treatment regimen was 29.8 months (interquartile range (IQR): 13.1–51.4); 83.8% of participants received at least one nucleoside reverse transcriptase inhibitor (NRTI), 70.1% at least one protease inhibitor (PI), and 23.6% at least one non-nucleoside reverse transcriptase inhibitor (NNRTI). At the time of assessment, 60.9% of the patients (131/225) had undetectable viral load and 64.4% (145/225) had no signs of severe immunosuppression (CD4 count > 350 cells/μL).

### 3.2. Association of Antiretroviral Treatment with Protection Against CMV Reactivation

#### 3.2.1. Humoral Response to CMV Measured by Specific IgG Levels

In total, 93.7% of the participants had specific IgG anti CMV antibodies. Anti-CMV IgG levels ranged from 1.2 to 19.5 IU/mL (median: 8.74 IU/mL; interquartile range (IQR): 5.6–10.9 IU/mL). None of the patients had CMV IgM antibodies. We observed a negative correlation between CMV IgG levels and current CD4 T-cell counts (rho = −0.21; *p* = 0.002), CD4/CD8 ratio (rho = −0.22; *p* = 0.001), and CD4 nadir count (rho = −0.34; *p* < 0.0001); additionally, CMV IgG titers showed a positive correlation with the time lengths spent with immunosuppression (CD4 < 200 cells/μL) (rho = 0.35; *p* < 0.0001). No association was recorded between CMV IgG and the current HIV viral load (rho = −0.04, *p* = 0.68) or cumulative time spent with detectable HIV RNA (rho = 0.05, *p* = 0.41), but a significant positive relationship was recorded between CMV IgG and HIV RNA zenith values (rho = 0.16, *p* = 0.02).

Regarding antiretroviral treatment, lower CMV antibody levels, indicating reduced CMV reactivation, were significantly associated with longer cumulative treatment duration (rho = −0.14, *p* = 0.03). No association was found between the time of exposure to current ARV regimen or the type of ARV regimen and CMV antibodies.

According to their response to treatment, the study participants were divided into the following groups: subjects with immunological treatment success/immunological treatment failure (CD4 > 350/< 350 cells/μL) and subjects with suppressed/non-suppressed HIV RNA levels (undetectable/detectable HIV plasma viral load).

**Figure 1:**
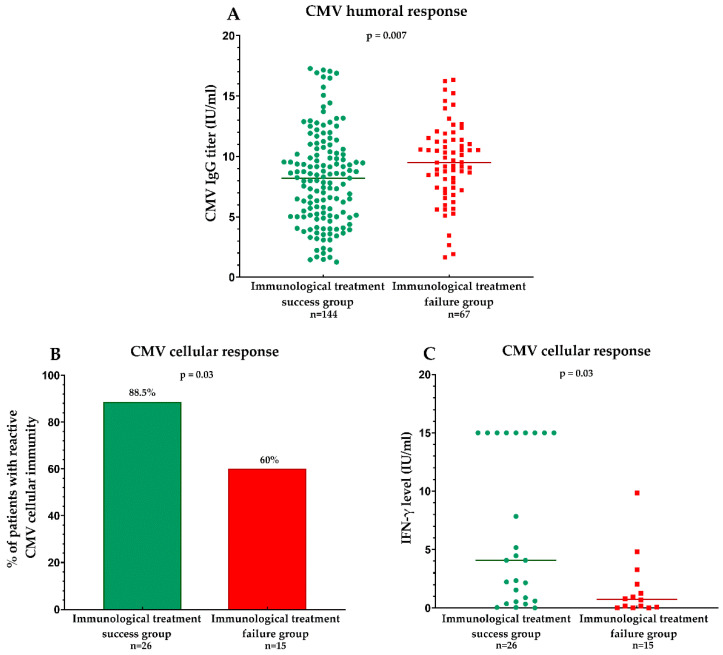
**Associations between immunological treatment success and CMV humoral and cellular immune response.** The humoral immune response to CMV was evaluated via anti-CMV IgG titers, while the cellular immune response was assessed using an interferon release assay to measure IFN-γ production by CD8+ T cells following CMV peptide stimulation. HIV-infected patients with immunological treatment success (CD4 > 350 cells/μL, red) exhibited significantly lower CMV IgG titers (Mann–Whitney U test) (**A**), a higher percentage of reactive cellular immunity (chi-squared test) (**B**), and higher IFN-γ levels (Mann–Whitney U test) (**C**) compared to patients without immune recovery (CD4 ≤ 350 cells/μL, green).

Patients with immunological treatment success had significantly lower CMV IgG titers compared to patients who failed to recover CD4^+^ T- cell counts (8.21 vs. 9.51 IU/mL; *p* = 0.007) (Figure 1A). The antiretroviral treatment impact on the CMV humoral immune response was similar in patients with/without active HIV replication: there were no significant differences in CMV IgG titers according to the level of HIV suppression (8.56 vs. 8.84 IU/mL in those with detectable and undetectable HIV viral load, respectively, *p* = 0.280).

#### 3.2.2. Cellular Response to CMV Measured by an Interferon Release Assay

In order to assess if an effective antiretroviral treatment is able to reconstitute a strong cellular immune response to CMV, we measured the interferon release by CD8+ T cells after stimulation with specific CMV peptides. The reconstitution of CMV-specific T cell responses was present in 78% of the tested participants, and the IFN-γ level ranged from 0.34 to 15 IU/mL (median: 4.07 IU/mL; interquartile range (IQR): 1.01–15 IU/mL). An effective T cell response was positively correlated with the CD4 (rho = 0.33, *p* = 0.03) and CD8-T cell counts (rho = 0.46, *p* = 0.002).

The proportion of subjects with reactive CMV-specific cellular immunity was significantly higher among patients with a good immunologic response to treatment vs. those who fail to recover CD4^+^ T-cell counts (88.5% vs. 60%; *p* = 0.03) (Figure 1B). A linear-by-linear association test reached statistical significance (*p* = 0.03), showing a strong association between CMV-specific cellular immunity and immune recovery after antiretroviral treatment. Furthermore, a significantly higher level of IFN-γ produced by CD8^+^ T-cells was recorded in the group of subjects with CD4^+^ T-cell immune recovery (4.4 vs. 2.07 IU/mL; *p* = 0.03) (Figure 1C).

We found no significant differences between patients with or without detectable HIV viral load in terms of CMV-specific cellular immune response (proportion of subjects with reactive CMV-specific cellular immunity: 77.3% vs.78.9%, *p* = 0.89; IFN-γ level: 8.49 vs. 9.16 IU/mL, *p* = 0.11).

### 3.3. Asymptomatic CMV Replication in Peripheral Blood Mononuclear Cells (PBMCs)

Only 17% of the patients had detectable CMV DNA with a median value of 12.6 (range 2.1–24.8) copies/million cells. Lower levels of CMV DNA were found among patients with a good immunologic response to treatment vs. those without immune recovery (proportion of subjects with detectable CMV DNA: 12% vs. 28.5%, *p* = 0.19; CMV DNA level: 0.87 vs. 5.14 copies/million cells, *p* = 0.34).

## 4. Discussion

We found that potent antiretroviral therapy (cART) with subsequent immunologic reconstitution may lead to effective control of CMV infection in a group of young HIV-positive adults who have lived with HIV infection almost their whole life. Patients with immunological success after therapy had significantly lower CMV IgG titers and higher levels of CMV-specific T-cell responses, compared to those with suboptimal immune recovery after cART.

The observed association between cART- induced immune restoration and CMV IgG levels is consistent with previous studies. Gómez-Mora et al. showed an elevated humoral response to cytomegalovirus, suggesting frequent asymptomatic reactivations, in HIV-infected individuals with poor CD4+ T-cell immune recovery [20]. High CMV IgG antibody levels were also found to be associated to a lower CD4+ response to antiretroviral therapy in a study of 81 HIV-infected women from sub-Saharan Africa [21]. It has been suggested that CMV infection may amplify the immunosenescent phenotype in PLWH infection, leading to lower levels of immunologic reconstitution, even in those who obtain suppression of viral replication [18].

Consistent with our findings, it has been shown that HIV-infected individuals on long-term ART tend to have higher CMV-specific T cells responses than those who have progressive disease or who have active CMV disease [22,23,24]. Bronke et al. have shown that IFN-γ-producing CD8^+^ T cells are detected at a higher level in samples from HIV-positive individuals who remained asymptomatic compared with samples from individuals progressing to AIDS [25] and, in the long term, successfully treated HIV-infected patients [26]. Stone et al. have demonstrated that effector memory and effector CMV-specific CD8+ T cells play an important role in controlling CMV infection in treated HIV-positive individuals [27].

We found a very low number of positive CMV DNA in our study group, with the level of CMV DNA being lower among patients with a good immunologic response to treatment, although the result did not reach statistically significance. Connick et al. have previously reported that immune reconstitution secondary to ART results in a significant and progressive decline in blood CMV viremia, even in the absence of specific anti-CMV therapy [28].

Taken together, these data suggest that successful antiretroviral treatment is effective in controlling a latent CMV infection, preventing reactivation in a group of young adults with long-term chronic HIV infection. As CMV and HIV transactivate each other, leading to a more profound immunosuppression [1], an effective antiretroviral treatment can trigger restoration of CMV-specific immune responses [29], after immune reconstitution. In turn, the specific CMV cellular immune response controls CMV viral replication and limits episodes of asymptomatic CMV reactivation [30]. In the long term, this may lead to reduced immune activation and inflammation, decreasing the risk of premature chronic comorbidities in HIV-infected patients.

## Figures and Tables

**Table 1:** HIV+ participant characteristics.

Characteristics of the Patients	Total (*n* = 225)
**Age,** median (years)	24 (23–25)
**Sex, males** (n; %)	52%
**Current CD4 count**, median (cells/μL)	476 (253–699)
**Cumulative time spent with CD4 < 200 cells/μL,** median (days)	372 (28.5–983)
**Current CD8 count**, median (cells/μL)	794 (571–1021)
**CD4/CD8 Ratio**, median	0.6 (0.3–0.9)
**Nadir CD4 count**, median (cells/μL)	92 (23–199)
**HIV RNA**	
HIV RNA, log10, median (copies/mL)	2.1 (0–4.1)
HIV RNA detectable (n; %)	94 (39.1%)
Cumulative time spent with detectable HIV RNA (days)	1575 (538–2774)
Zenith HIV RNA, log10, median (copies/mL)	5.1 (4.4–5.6)
**Estimated duration of HIV**, median (years)	23.7 (22.8–24.6)
**Antiretroviral treatment**	
Total months of exposure to ARVs, median	139.6 (96.4–172.6)
Months of exposure to current ARV regimen, median	29.8 (13.1–51.4)
Any PI use (current) (n; %)	70.1%
Any NRTI use (current) (n; %)	83.8%
Any NNRTI use (current) (n; %)	23.6%
**Positive for CMV IgG antibodies** (n; %)	211 (93.7%)
**QuantiFERON-CMV-reactive** (n; %), n = 41	32 (78%)
**CMV DNA detectable** (n; %), n = 41	7 (17%)

Values are expressed as median (IQR) or as percentages.

## Data Availability

The raw data supporting the conclusions of this article will be made available by the authors on request.
